# CRISTOPH – A cluster-randomised intervention study to optimise the treatment of patients with hypertension in General Practice

**DOI:** 10.1186/1471-2296-9-33

**Published:** 2008-06-10

**Authors:** Achim Mortsiefer, Tobias Meysen, Martin Schumacher, Claudia Lintges, Maren Stamer, Norbert Schmacke, Karl Wegscheider, Heinz-Harald Abholz, Jürgen in der Schmitten

**Affiliations:** 1 Department of General Practice, University Hospital, P.O. Box 101001, 40225 Düsseldorf, Germany; 2 Health Systems Research Chair (AKG), University of Bremen, Bibliothekstr. 1, 28359 Bremen, Germany; 3 Department of Medical Biometry and Epidemiology, University Medical Center Hamburg-Eppendorf, Martinistrasse 52, 20246 Hamburg, Germany

## Abstract

**Background:**

Recent guidelines for the management of hypertension focus on treating patients according to their global cardiovascular risk (CVR), rather than strictly keeping blood pressure, or other risk factors, below set limit values. The objective of this study is to compare the effect of a simple versus a complex educational intervention implementing this new concept among General Practitioners (GPs).

**Methods/design:**

A prospective longitudinal cluster-randomised intervention trial with 94 German GPs consecutively enroling 40 patients each with known hypertension. All GPs then received a written manual specifically developed to transfer the global concept of CVR into daily General Practice. After cluster-randomisation, half of the GPs additionally received a clinical outreach visit, with a trained peer discussing with them the concept of global CVR referring to example study patients from the respective GP. Main outcome measure is the improvement of calculated CVR six months after intervention in the subgroup of patients with high CVR (but no history of cardiovascular disease), defined as 10-year-mortality ≥ 5% employing the European SCORE formula. Secondary outcome measures include the intervention's effect on single risk factors, and on prescription rates of drugs targeting CVR. All outcome measures are separately studied in the three subgroups of patients with 1. high CVR (defined as above), 2. low CVR (SCORE < 5%), and 3. a history of cardiovascular disease. The influence of age, sex, social status, and the perceived quality of the respective doctor-patient-relation on the effects will be examined.

**Discussion:**

To our knowledge, no other published intervention study has yet evaluated the impact of educating GPs with the goal to treat patients with hypertension according to their global cardiovascular risk.

**Trial registration:**

ISRCTN44478543

## Background

### Hypertension management and global cardiovascular risk

Arterial hypertension is a major risk factor for cardiovascular diseases (CVD) such as myocardial infarction and stroke. The detection and treatment of elevated blood pressure (BP) and the management of patients with hypertension is an important challenge of daily practice in primary care. Previous studies found that only 60% of patients with known hypertension receive treatment, and in fewer than 50% of treated patients BP was controlled below 140/90 mmHg [1-3].

However, recent guidelines on hypertension [4-6], and on both primary and secondary cardiovascular prevention [7] emphasise that rather than to focus on single risk factors, intensity of care should focus on the global cardiovascular risk (CVR) of an individual patient. Global cardiovascular risk is an arithmetical compound of age, sex, and the known cardiovascular risk factors, and can be approximated by means of various risk calculators. If pharmacological risk factor treatment promises a relative risk reduction of some 25% [8,9], or possibly up to 80% in combination therapy [10], then a *relevant *absolute risk reduction is only possible where there is a relevant absolute risk to begin with. According to the concept of global cardiovascular risk, therefore, pharmacological treatment is only recommended for patients with a cardiovascular 10-year-mortality at or above 5%. The essence of this paradigmatic change is that treatment efforts and resources should be concentrated on where the (high) cardiovascular risk is: "treat risk, not risk factors" [11]. For general practitioners (GPs), this concept is of particular interest because it firstly focuses efforts and resources on high-risk patients, secondly makes GPs and patients more flexible in the choice of risk-lowering interventions, and thirdly reassures patients with low CVR that they may not benefit from treatment despite possible elevated risk factor levels.

### Implementing the concept of global CVR for the treatment of patients with hypertension

Few studies have been reported [12,13] addressing hypertension management in primary care with explicit consideration of global cardiovascular risk, none of them from Germany, and none of them measuring CVR before and after the intervention. The little available data suggest that GPs are not familiar with the new concept of global cardiovascular risk [14,15].

Initiating a process of behavioural change concerning guideline adherence of physicians is a challenge. Systematic reviews of interventions to change professional practice show that passive dissemination of information has little or no effect [16]; combined interventions using audits and feedbacks yield a larger impact, and clinical outreach visits seem to bring about the largest observed changes [17]. Outcomes are better if interventions include active and passive elements of learning, and if they use individual face-to-face interactions [18].

### Objective and research questions

Our general objective is to demonstrate that GPs who have been made familiar with the concept of global CVR by means of a complex, tailored intervention will adapt their treatment accordingly, resulting in measurable changes in patients' clinical outcomes. In particular, these are our questions:

1. In a subgroup of patients with high CVR (but no history of CVD), does a complex educational intervention directed at the GPs, including an educational outreach visit, lead to a higher improvement of mean CVR than a passive intervention, i.e. posting a manual?

2. What are the differential effects of the two interventions on various clinical targets in three risk-defined subgroups of patients with hypertension, namely patients with (i) a history of CVD, (ii) high CVR, but no history of CVD, and (iii) low CVR?

3. Are the results influenced by patient age, sex, or social status, or by the perceived quality of the respective doctor-patient-relationship?

In order to gain a deeper understanding of possible outcomes and their background, we also conducted an embedded qualitative study, employing semi-structured expert interviews in order to explore GPs' views and attitudes towards the new global CVR concept. The corresponding study protocol and its results will be published elsewhere.

## Methods/Design

### Ethics and Registration

Approval was granted by the Ethics Committee of the medical faculty of the University of Duesseldorf (no. 2715). Patient consent was not deemed necessary by the Committee since we use anonymous data routinely collected by GPs.

The trial was registered at ISRCTN44478543.

### Design

Prospective non-blinded longitudinal cluster-randomised intervention study. One GP (or one group practice with 1–3 GPs, respectively) represents a cluster, whereas the patients are the observation units.

After enrolling the patients, GPs were cluster-randomised into two groups, A and B. GPs of group A received a written manual by mail explaining the concept of global CVR. In addition, the GPs of this group received a tailored intervention including a clinical outreach visit (for details cf. *Intervention*). GP's of group B received only the written manual by mail with no further educative intervention.

Thus, the complex intervention (group A) is not compared to usual care, but to a low-level intervention (group B). This reflects our hypothesis that the benefit of the complex and therefore more-costly intervention may be relevant only if the effect can be demonstrated against a less-complex, low-cost intervention.

**Figure 1:**
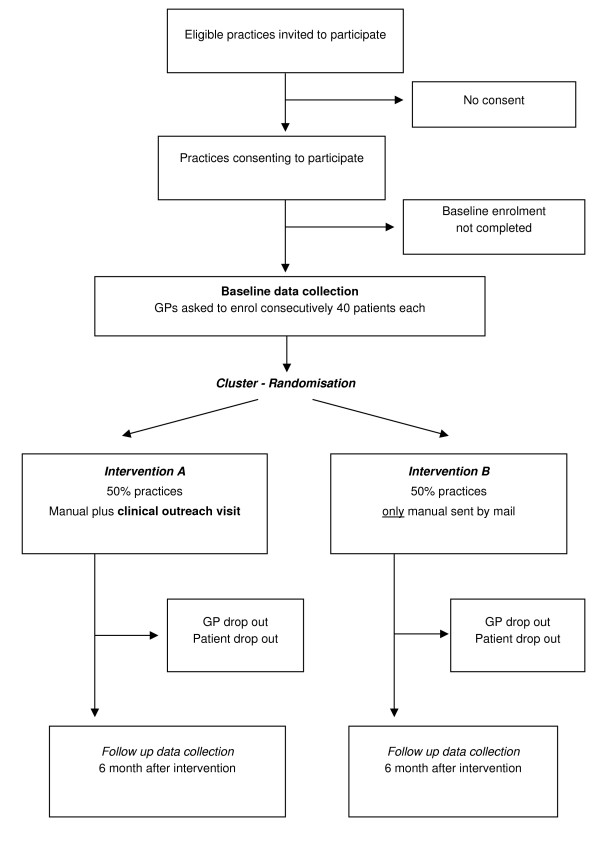
Flowchart.

Figure 1 is a flow chart of this two-group intervention trial.

### Calculation of cardiovascular risk

Global CVR is calculated employing the European SCORE formula, modified by factor 2 or 3 for men and women, respectively, with diabetes. We follow the SCORE working group's definition of a 10-year cardiovascular *mortality *< 5% for "low" risk, and ≥ 5% for "high" risk in patients with no history of cardiovascular disease, corresponding with the 20% dividing line commonly used in cardiovascular *morbidity *tables [19]. We opt for the SCORE rather than for the Framingham or PROCAM [20] risk tables because they are based on data for both men and women, and retrieved from recent national (German) cohort studies.

### Anticipated dropouts and sample size calculation

Sample size calculation refers to research question 1 (see above). In order to demonstrate an effect of reducing mean calculated CVR by a tenth in the subgroup of patients with high CVR (SCORE ≥ 5%, no history of cardiovascular disease) with a power of 80%, we calculated a necessary initial sample size of 2 × 51 recruited GPs enrolling 40 patients each. We estimated a GP drop out during the enrolment phase of 5%, a patient enrolment rate of 95%, a GP drop out between baseline and follow up of a further 5%, and a patient drop out between baseline and follow up of 10% (resulting in 3146 patients enrolled by 92 GPs). The sample size calculation was based on the assumption of a standard deviation of 0.44 for the CVR changes on the log-odds scale, an intra-class correlation of 0.2 and a proportion of 40% high risk patients in the total sample, the latter derived from a pilot study with 330 patients cared for by 20 GPs. According to these assumptions, 2 × 816 patients were required for the primary analysis in the high risk group.

### Participants and randomisation

We recruited GPs in the district of North-Rhine, choosing three circumscribed regions close to the cities of Düsseldorf, Cologne, and Aachen, one region for each of the three attending peers (convenience sample).

A random sample of the registered GPs in a given postal code were invited to participate by fax, and called 3–5 days later by one of the three trained peers. Incentives to participate were 1. the offer of a free and pharma-independent manual and, depending on randomisation, possibly peer training in a relevant field of General Practice; 2. the opportunity to take part in an innovative research project developed by GPs for GPs; and 3. an honorarium of € 300 for completing both data surveys (requiring a net time investment of 5 to 6 hours).

After patient enrolment and baseline data collection, the GPs (clusters) were randomised to intervention groups A and B.

In order to avoid the possibility that GPs already familiar with the concept of global CVR were accidentally over-represented in either group, we asked the GPs to estimate the individual CVR of each patient they enrolled. We compared this estimate with the respective patients' calculated CVR, and divided the GPs into two groups, according to whether their estimate is close to or far from the calculated CVR. We assumed this division to approximate a measure for *familiarity with the concept of global CVR*, and for randomisation, GPs were stratified by this attribute.

### Patient enrolment

Every GP was asked to enrol from his or her daily patient flow a consecutive sample of 40 patients with a known diagnosis of hypertension, regardless of the cause for consultation. Further inclusion criteria were age 40–75 years, and continuity of care by this GP over at least six months. Emergency cases were excepted, and patients expected to die within 1 year were not included.

### Intervention

In order to address the complex issue of hypertension management in the light of absolute cardiovascular risk, we developed a multi-faceted intervention through a process of identifying barriers to implementation, e.g. comprehensibility of the new concept, and communication pitfalls. We compiled a written manual (17 pages) on the basis of the ESC-Guidelines [7] with the following chapters:

1. Introduction.

2. What is new about the "concept of global CVR?"

3. How to determine global CVR?

4. What is the likely individual benefit of the treatment of cardiovascular risk factors?

5. Two examples of decision-making.

6. Non-pharmacological therapy: motivation for behavioural change and psychosocial support.

7. Pharmacologic treatment with antihypertensive drugs.

8. Pharmacologic treatment with lipid-lowering drugs.

9. Literature.

This manual was sent by mail to all GPs (randomisation groups A and B). In addition, GPs in group A received a personal intervention including an outreach visit by a peer (30–45 min.), a feedback telephone call after three weeks (5–10 min.), and delivery of a block of 50 patient information sheets. Thus, we compared the effect of an elaborate intervention (A) with the conventional process of postal dissemination of a guideline-based manual (B), which is generally assumed to have no or little significant effect on physician behaviour [16].

During the outreach visit, peer and GP discussed the new concept on the basis of (a) our manual and (b) 3–4 suitable cases selected by the peer from this GP's baseline data. The cases were chosen to include at least one patient with a history of CVD, and at least two patients without such a history; of the latter one at low risk (SCORE < 5%), and one at high risk (SCORE > 5%), respectively. The discussion of the GPs' respective patient cases included feedback elements, and served as concrete examples to demonstrate the paradigmatic changes of the CVR concept to the GP. Furthermore, the GPs were instructed how to use the SCORE calculator of global cardiovascular risk (print version, included in the manual), and additional patient information sheets.

Three practising GPs (TM, AM, and JidS) were trained to explore GPs' understanding, beliefs, and attitudes during the initial phase of the conversation in order to tailor the concept's message to the individual colleague they were talking to. The process of standardisation was facilitated by a dialogue draft for the outreach visit, regular audit-meetings, and personal feedback from a passive observer who attended the first three visits of each peer.

### Our intervention focused on five key messages

1 Therapeutic decisions in the management of hypertension should always be preceded by an estimation of absolute CVR.

2 Within certain limits, there are no fixed targets for blood pressure or cholesterol level. Rather, the attainable absolute risk reductions by lowering blood pressure or taking a statin depend on the absolute CVR before treatment.

3 All patients with manifest cardiovascular diseases (CVD) are candidates for intensive treatment because of their very high recurrence risk for CVD. In primary prevention, risk factor treatment should intensify with rising CVR, whereas patients with low CVR (defined by SCORE < 5%) have no or little proven benefit from treatment.

4 There are frequently several options to reduce cardiovascular risk. If pharmacological treatment of one risk factor remains unsatisfactory, e.g. refractory hypertension or intolerable side effects of anti-hypertensive drugs, then other options should be considered, such as prescription of a statin in this patient regardless of a "normal" cholesterol level.

5 The explanation of individual CVR is meant to enhance communication between physician and patient, represents an opportunity to invite the patient to take part in a process of shared decision making, and can be a potentially powerful vehicle to promote patient autonomy.

### Data collection

Patient enrolment and recording of baseline data took place before randomisation. Follow up data collection was conducted 6 to 9 months after the intervention. We employed a self-developed paper documentation because of the limited extent and availability of routine computerised data.

At baseline, the documentation on each patient included age, sex, smoking habits, and history of one or more of the following: diabetes mellitus, nephropathy, and manifest cardiovascular disease (namely, coronary artery disease, cerebrovascular disease, and peripheral artery disease). In addition, the GPs were requested to indicate drugs relevant for cardiovascular disease, i.e. antihypertensive, lipid-lowering, and antiplatelet drugs. The actual blood pressure reading (at enrolment) and the latest cholesterol measurement (before enrolment) were also noted.

At baseline, GP's were also asked to judge the perceived quality of the doctor-patient-relationship for each patient, and to estimate the patient's CVR (as a 10-year-mortality-risk) on a Likert scale of 1–5.

In addition, we issued a patient questionnaire at baseline to all enrolled patients asking for CVD family history, education, physical training, perceived quality of doctor-patient-relation, personal interest in taking part in treatment decisions, and two questions concerning their knowledge of global CVR.

At follow-up, actual levels of blood pressure and cholesterol were documented by practice nurses. New onset of diabetes mellitus, nephropathy, or cardiovascular events were recorded. GP's were requested to note any changes in cardiovascular medical treatment. Smoking status was updated.

### Data Retrieval and Monitoring

Every GP received instruction on study procedures individually by one of the three peers. At baseline, data sheets were directed to GPs only, but at follow-up there were different data sheets for practice nurse and GP, respectively, restricting the workload of the GPs to data only they could provide. Data sheets were personally collected by the peers at baseline, and sent by mail by the GPs at follow-up. Data were entered into electronic sheets by medical students. Practice nurses or, where necessary, GPs were contacted if inconsistencies occurred or to complete missing data.

In order to monitor the quality of the data submitted by the GPs at baseline, at the end of the study we issued a data sheet to the practice nurses requiring them to re-enter the baseline data for blood pressure and cholesterol from the electronic record. Since the written baseline data sheets had been collected from the GPs, we regarded it as unlikely that the nurses or GPs would recall after follow up what numbers had been entered at baseline. We will examine the congruence of the data documented by the GPs on the baseline forms with the data referring to the same calendar day entered by the practice nurses some 6–9 months later.

### Incentives for participation

At recruitment, each GP was offered compensation of € 300 for an estimated total workload of at least 6 hours, plus € 40 for the responsible practice nurse. In addition, practice nurses involved in the monitoring process received € 15 each.

### Outcome measures

We will calculate changes in global CVR, mean blood pressure, total cholesterol, smoking rate, blood pressure control rate, and prescription rates of CVR-lowering drugs (ASS and statins).

These outcome measures are to be calculated separately for three different subgroups of patients defined by their CVR:

Group I: Patients with a history of manifest cardiovascular disease (CVD).

Group II: Patients with high CVR (SCORE ≥ 5%), no history of CVD.

Group III: Patients with low CVR (SCORE < 5%), no history of CVD.

### Statistical analysis

The primary analysis includes all randomised patients of group II with global CVR determinations at baseline (ITT population). CVR values at baseline as well as at follow-up will be transformed on the log-odds scale (multiplicative model). In case of missing follow-up values, a LOCF (last-observation carried forward) imputation will be performed, i.e. the baseline determination will be imputed as follow-up determination. For the primary analysis, a two-level random coefficient model will be fitted to the data with patients nested in physicians modeled as random effects. This model takes the correlation structure resulting from cluster randomisation into account and allows for differences between physicians in treatment effects. The primary analysis will compare the baseline-adjusted follow-up means between random groups at a significance level of 0.05, two-sided.

As sensitivity analyses, calculations will be repeated for the complete cases population and for a dataset with missing values imputed using an EM algorithm.

Secondary analyses will be in the complete cases only. Analogous to the primary analysis, mixed models will be fitted for the secondary endpoints in group I, group II and group III separately.

In the exploratory part of the analysis, the covariates age, sex, social status, and perceived quality of the respective doctor-patient-relationship will be added as confounders and potential effect modifiers to the statistical model. By backward selection, optimal models will be identified and reported.

Calculations will be performed with SPSS and STATA, last available versions.

## Discussion

In the past, intervention studies to improve the treatment of patients with hypertension in General Practice have focused on educating GPs on how to achieve a maximum reduction of blood pressure regardless of individual CVR [21]. Our intervention, in contrast, specifically addresses patients' individual CVR and may thus lead to varying therapeutical approaches in patients with different risks, despite identical blood pressure readings.

## Abbreviations

BP: blood pressure; CVD: cardiovascular disease; CVR: cardiovascular risk; EM: expectation maximisation; ESC: European society of cardiology; GP: general practitioner; ITT: intention to treat; LOCF: last observation carried forward; PROCAM: Prospective Cardiovascular Muenster Study; SCORE: systematic coronary risk evaluation.

## Competing interests

The authors declare that they have no competing interests.

## Authors' contributions

HHA, NS and KW conceived the study concept. JidS and AM carried out the study, supported by TM, MS, and CL. AM and JidS drafted the manuscript. KW calculated the sample size, and developed the concept for biostatistical analysis. NS and MS participated in the development of the study design. All authors read and approved of the final manuscript.

## Pre-publication history

The pre-publication history for this paper can be accessed here:


